# Draft Genome Sequence of a Putatively Antibiotic-Resistant Escherichia coli Strain Isolated from Calf Intestine in Boyacá, Colombia

**DOI:** 10.1128/mra.01100-22

**Published:** 2023-01-25

**Authors:** Maria Inés Torres Caycedo, Javier Rodríguez López, Rubén Pérez Pulido, Rosario Lucas, Antonio Galvez, Maria José Grande Burgos

**Affiliations:** a Facultad de Ciencias de la Salud, University of Boyacá, Tunja, Colombia; b Department of Health Sciences, University of Jaén, Jaén, Spain; Loyola University Chicago

## Abstract

A draft genome of the putatively antibiotic-resistant Escherichia coli strain ANGUJ1, which was isolated from calf intestine from Boyacá, Colombia, is reported. The genome possessed genetic determinants for antibiotic resistance and multicompound resistance efflux pumps. *In silico* prediction analysis suggests phenotypic resistance to six classes of antibiotics plus aldehyde and peroxide.

## ANNOUNCEMENT

Cow intestine is consumed in Colombia as a food, either boiled or grilled, and is used as casing for fermented meats. There is a risk of transmission of antibiotic-resistant Escherichia coli from cow intestine either by cross-contamination or by consumption of undercooked intestine portions. Disinfection resistance may improve the survival of E. coli on food contact surfaces and utensils.

E. coli strain ANGUJ1 was isolated from raw cow large intestine sold over the counter in a local food market in Boyacá, Colombia. The sample (5 g) was separated aseptically with a sterile knife, homogenized in 45 mL sterile saline solution for 5 min (Stomacher 400; Seward, UK), and plated on MacConkey agar (Millipore, Darmstadt, Germany) at 37°C for 24 h. A single colony was repurified on MacConkey agar. An overnight culture (1.5 mL in tryptic soy broth [TSB] at 37°C) was centrifuged for 5 min at 13,500 rpm in a microcentrifuge (Eppendorf, Madrid, Spain), and the cell pellet was used for extraction of total DNA (GenElute bacterial genomic DNA extraction kit; Sigma-Aldrich, Munich, Germany). E. coli identity was confirmed by PCR amplification of the 16S rRNA gene with 27F and 1492R primers (1) and sequencing. The extracted DNA was also used for sequencing of the bacterial genome. DNA libraries were prepared following the DNA preparation reference guide (2), with a DNA preparation kit (reference 20060059; Illumina). Input DNA was diluted to 0.2 ng/μL to start the protocol. At the multiplexing step, Nextera DNA combinatorial dual (CD) indexes (reference 20018708; Illumina) were used. Library size selection was performed according to the Illumina library preparation protocol, with sample purification beads (SPB) included in the library preparation kit. Library size was validated by using the Fragment Analyzer 48-capillary array with the high-sensitivity (HS) next-generation sequencing (NGS) fragment kit (1 to 6,000 bp) (reference DNF-474-0500; Agilent). The libraries were sequenced with a NextSeq 2 × 150-bp paired-end reagent kit (NextSeq 500/550 mid-output kit v2.5, reference 20024905) on a NextSeq 500 sequencer, according to the manufacturer’s instructions. Quality assessment was performed with the fastp program (3), applying the following parameters: min_length: 50; trim_qual_right: 30; trim_qual_type: mean; trim_qual_window: 10. The assembly was carried out with SPAdes v3.15.5 (4) and Pilon v1.24 (5) with default parameters to improve the draft assembly. Open reading frame identification and gene annotation were carried out using the Bakta annotation pipeline v1.12 (6) (Table 1).

**TABLE 1:** Basic characteristics of the genome assembly of the E. coli BC40 strain

Parameter	Finding
No. of reads	15,250,398
Assembly size (bp)	4,795,503
No. of contigs	129
G+C content (%)	50.5
*N*_50_ (bp)	137,552
No. of coding sequences	4,467
Total no. of RNAs	111
Avg. coverage (×)	65.082

The *in silico* antibiogram (ResFinder v4.1.11 [7], run with default parameters) indicated phenotypic resistance to six antibiotic classes (aminoglycosides [*aadA1*], quinolones [*qnrB19*], β-lactams [*bla*_TEM1-C_], folate pathway antagonists [*sul3* and *dfrA1*], tetracyclines [*tet*(A)], and amphenicols [*floR*]), aldehyde (*formA*), and peroxide (*sitABCD*).

### Data availability.

Data have been deposited in the ENA database. The accession numbers are GCA_947310805 (draft genome assembly) and ERS13459498 (raw reads).
